# Partitioning of Interstitial Segregants during Decohesion: A DFT Case Study of the Σ3 Symmetric Tilt Grain Boundary in Ferritic Steel

**DOI:** 10.3390/ma12182971

**Published:** 2019-09-13

**Authors:** Xiang Huang, Rebecca Janisch

**Affiliations:** 1 Interdisciplinary Centre of Advanced Materials Simulation (ICAMS), Ruhr-University Bochum, 44780 Bochum, Germany; 2 Department of Chemistry, Technical University of Munich, 85747 Garching, Germany

**Keywords:** Hydrogen embrittlement, grain boundary segregation, ab-initio calculations, decohesion

## Abstract

The effect of hydrogen atoms at grain boundaries in metals is usually detrimental to the cohesion of the interface. This effect can be quantified in terms of the strengthening energy, which is obtained following the thermodynamic model of Rice and Wang. A critical component of this model is the bonding or solution energy of the atoms to the free surfaces that are created during decohesion. At a grain boundary in a multicomponent system, it is not immediately clear how the different species would partition and distribute on the cleaved free surfaces. In this work, it is demonstrated that the choice of partitioning pattern has a significant effect on the predicted influence of H and C on grain boundary cohesion. To this end, the $\Sigma 3\left(112\right)\left[1\bar{1}0\right]$ symmetric tilt grain boundary in bcc Fe with different contents of interstitial C and H was studied, taking into account all possible distributions of the elements, as well as surface diffusion effects. H as a single element has a negative influence on grain boundary cohesion, independent of the details of the H distribution. C, on the other hand, can act both ways, enhancing or reducing the cohesion of the interface. The effect of mixed H and C compositions depends on the partition pattern. However, the general trend is that the number of detrimental cases increases with increasing H content. A decomposition of the strengthening energy into chemical and mechanical contributions shows that the elastic contribution dominates at high C contents, while the chemical contribution sets the trend for high H contents.

## 1. Introduction

The mechanism of hydrogen enhanced decohesion (HEDE) along grain boundaries in iron and high-strength steel has been investigated in various experimental studies (e.g., [1,2]), but a clear picture is still under cover. The effect can be promoted [3] or hindered [4] by the segregation of alloying elements, but the experimental results are sometimes contradicting [3,5], and difficult to predict in a multi-component system [6]. This study focused on carbon, as the inevitable alloying element in steel, and investigated its combined effect with H on a $\Sigma 3\left(112\right)\left[1\bar{1}0\right]$ symmetric tilt grain boundary (STGB). A higher percentage of special grain boundaries (primarily twin) in microstructures is commonly assumed to enhance the tensile ductility and toughness of metallic microstructures charged with H [7] by reducing grain boundary segregation and embrittlement. However, other studies point to such coherent twin boundaries as the microstructural features most susceptible to crack initiation [8].

Ab-initio density functional theory (DFT) [9,10] is a method widely applied in physics and chemistry, especially in investigations of properties of molecules and solid state materials, to solve the many-body Schrödinger equation [11]. DFT calculations provide the possibility of a quantitative determination of the segregation and trapping energies of H at various defects [12] and of the work of separation (WoS) and cohesive strength of interfaces with different structure and composition [13,14]. The typical computational strategy is to separate a grain boundary supercell into two free surfaces by a quasistatic, stepwise procedure, which gives rise to different partition patterns of the segregating atoms on the cleaved free surfaces. Commonly, it is assumed that the system is in thermodynamic equilibrium, which means that the work of separation is determined for the surface configuration with the lowest energy. In the process of crack nucleation and propagation, however, this would mean that the segregated atoms have enough time to interchange between the two newly created surfaces to establish this equilibrium. This might be a realistic assumption for H, but not so for C. In this work, it is demonstrated that a different partitioning of segregants can lead to a different prediction of their effect on grain boundary cohesion.

Tahir et al. [15] found for the Σ5(310)[001] grain boundary that the carbon-segregated interface should be considered as the ground state of this grain boundary in any Fe-C alloy. This in turn leads to a better understanding of HEDE. Carbon significantly decreases the grain boundary energy and increases the work of separation, where around 90% is contributed by the chemical interaction of C with the bcc Fe host. HEDE can then be understood as the replacement of C with H, i.e., a co-doping of C and H at the grain boundary. The segregation energy in [15] shows that this is a likely scenario, and that it causes a detrimental mechanical contribution to the grain boundary and weakens chemical binding between C and its Fe nearest neighbors. In a second study, Wang et al. [16] showed that the solution energy of carbon exhibits similar trends at different grain boundaries (at Σ5(310), Σ5(210) and Σ3(112) GBs), namely that at a low areal density (up to roughly 0.04/Å^2^) the solution energy is constant or changes only slightly, i.e., the atoms at the interface do not interact. Above this concentration (for more than 50% occupation of the interstitial sites), the solution energy increases. The same interaction that leads to this increase could cause the above-mentioned co-segregation effect also at the Σ3(112) GB, which is investigated further in this paper.

The paper is organized as follows. Firstly, the basic equations from which the grain boundary properties are obtained are introduced in Section 2, and then the computational details are presented in Section 3. The results for grain boundary, solution, and segregation energies are presented in Section 4.1, Section 4.2 and Section 4.3, and, finally the work of separation for different partition patterns at the (112) surfaces is discussed, along with the chemical and mechanical contribution of the interstitials to the change in work of separation compared to the GB in pure Fe (Section 4.4). The paper finishes with a critical discussion of the results (Section 5) and conclusions are drawn in Section 6.

## 2. Background

According to the thermodynamic theory of Rice and Wang [17], the tendency of an impurity to segregate to a free surface (FS) instead of to the grain boundary (GB) is characteristic for a detrimental influence of the element on grain boundary cohesion. This can be quantified with DFT calculations [18]. Here, the strengthening energy, also often called bonding energy difference, is defined as follows (Note that compared to the original work of Rice and Wang, the sign in Equation (1) is reversed. This definition has the advantage that it leads to Equation (9), where a positive sign means that the segregating atom increases the work of separation.):

(1) $$\begin{array}{c} \Delta E_{\mathrm{SE}}=E_{B,\mathrm{GB}}-E_{B,\mathrm{FS}}=E_{\mathrm{seg},\mathrm{FS}}-E_{\mathrm{seg},\mathrm{GB}}, \end{array}$$

with the bonding energy $E_{B,S}$ to an interface *S* (GB or FS)

(2) $$\begin{array}{c} E_{B,S}=\frac{E_{\mathrm{tot},S}+\mu_{X}-E_{\mathrm{tot},S}^{X}}{2A}. \end{array}$$

$E_{\mathrm{tot},S}$ with/without superscript *X* denotes the total energy containing interface *S* with/without the segregating element *X*. The chemical potential $\mu_{X}$ equals to the energy of the segregating element in its stable reference bulk phase. The bonding energy, Equation (2), and hence the strengthening energy, too, is normalized with the interface area A. The factor of two arises due to the periodic boundary conditions in the calculation. As indicated in Equation (1), the strengthening energy can also be expressed via the segregation energies, $E_{\mathrm{seg},S}$. The segregation energy of interstitial atoms to an interface *S* is defined as the difference between their solution energy at the interface and in perfect bulk, again normalized with the interface area.

$$\begin{array}{c} E_{\mathrm{seg},S}=\frac{1}{2A}\left[E_{\mathrm{sol},S}^{aC,bH}-E_{\mathrm{sol},\mathrm{bulk}}^{aC,bH}\right]. \end{array}$$

Here, *a* means the number of segregating C and *b* the number of segregating H interstitial atoms.

The solution energy of an atom at an interface, or in the bulk crystal, can be defined as the difference between the total energy of the structure models with the atom, $E_{\mathrm{tot},S}^{nC,mH}$, and the structure models with one fewer atoms plus the atom’s chemical potential μ:

(3) $$\begin{array}{ccc} E_{\mathrm{sol},S}^{aC,bH} & = & \frac{1}{\left|a|+|b\right|}\left[E_{\mathrm{tot},S}^{nC,mH}-E_{\mathrm{tot},S}^{(n-a)C,(m-b)H}-a\mu_{C}-b\mu_{H}\right]. \end{array}$$

In this case, *S* labels the grain boundary (GB), the free surface (FS), or the bulk single crystal. *n* (*m*) is the actual number of C (H) atoms in the system, after single atoms have been added or replaced, respectively, in the following manner: the C concentration is initially increased step by step, starting from the pure Fe STGB. In this case, *a* will be either 1 in a sequential solution scheme, or equal to *n* in a concurrent scheme, while *b* is zero. Afterwards, once full occupation of the GB with C is reached (with four interstitial atoms per GB), the C atoms are one by one replaced by H, until the GB or surface is fully occupied by H. In this case, *a* is either −1 in the sequential scheme (*b* = 1) or equal to *n* in the concurrent scheme (b=m).

The general expression for the segregation energy, of which the concurrent and sequential scenario are special cases, then becomes

(4) $$\begin{array}{c} E_{\mathrm{seg},\mathrm{GB}}=\frac{1}{2A}\frac{1}{\left|a|+|b\right|}\left[E_{\mathrm{tot},\mathrm{GB}}^{nC,mH}-E_{\mathrm{tot},\mathrm{GB}}^{(n-a)C,(m-b)H}-E_{\mathrm{tot},\mathrm{bulk}}^{nC,mH}+E_{\mathrm{tot},\mathrm{bulk}}^{(n-a)C,(m-b)H}\right]. \end{array}$$

In a concurrent scheme, the reference structure is always the pure Fe STGB: a=n,b=m. Then, Equation (4) reduces to

(5) $$\begin{array}{c} E_{\mathrm{seg}\_\mathrm{con},\mathrm{GB}}=\frac{1}{n+m}\frac{1}{2A}\left[E_{\mathrm{tot},\mathrm{GB}}^{nC,mH}-E_{\mathrm{tot},\mathrm{GB}}-E_{\mathrm{tot},\mathrm{bulk}}^{nC,mH}+E_{\mathrm{tot},\mathrm{bulk}}\right]. \end{array}$$

In the sequential scheme, two regimes are distinguished, as mentioned above: (1) increasing the C concentration at the otherwise pure Fe GB step by step; and (2) subsequent replacement of C by H. In both cases, the actual composition differs from the previous one by one atom. In the first regime, (a=1,b=m=0), Equation (4) thus becomes

(6) $$\begin{array}{c} E_{\mathrm{seg}\_\mathrm{seq}.\mathrm{GB}}^{+C}=\frac{1}{2A}\left[E_{\mathrm{tot},\mathrm{GB}}^{nC}-E_{\mathrm{tot},\mathrm{GB}}^{(n-1)C}-E_{\mathrm{tot},\mathrm{bulk}}^{nC}-E_{\mathrm{tot},\mathrm{bulk}}^{(n-1)C}\right],\;\left[n=1,\cdots ,4\right]. \end{array}$$

For the subsequent stepwise replacement of C with H (a=−1,b=1). it becomes

(7) $$\begin{array}{c} E_{\mathrm{seg}\_\mathrm{seq},\mathrm{GB}}^{-C,+H}=\frac{1}{2\cdot 2A}\left[E_{\mathrm{tot},\mathrm{GB}}^{nC,mH}-E_{\mathrm{tot},\mathrm{GB}}^{(n+1)C,(m-1)H}-E_{\mathrm{tot},\mathrm{bulk}}^{nC,mH}-E_{\mathrm{tot},\mathrm{bulk}}^{(n+1)C,(m-1)H}\right],\;\left[n=3,\cdots ,0;m=1,\cdots ,4\right]. \end{array}$$

Coming back to the strengthening energy, if $\Delta E_{\mathrm{SE}}<0$, the impurity element decreases GB cohesion, and, if $\Delta E_{\mathrm{SE}}>0$, it enhances GB cohesion. This can be understood by inserting Equation (2) into Equation (1) and re-arranging the terms to

(8) $$\begin{array}{ccc} \Delta E_{\mathrm{SE}} & = & \frac{\left(2E_{\mathrm{tot},\mathrm{FS}}^{X}-E_{\mathrm{tot},\mathrm{GB}}^{X}\right)-\left(2E_{\mathrm{tot},\mathrm{FS}}-E_{\mathrm{tot},\mathrm{GB}}\right)}{2A} \end{array}$$

(9) $$\begin{array}{ll} = & WoS^{X}-WoS, \end{array}$$

which means that the strengthening energy equals the difference in work of separation with and without impurity at the interface. Note that the bonding energy at the surface enters Equation (9) twice, in order to preserve the number of atoms. According to Griffith [19], crack propagation along a cleavage plane (or grain boundary) will take place if the elastic strain energy in the system compensates the energy that is needed to create two new surfaces. In the case of a grain boundary, this energy equals the work of separation. Hence, a reduction of WoS, i.e., a negative strengthening energy, or bonding energy difference, indicates a reduction in cohesion. Following the approach of Geng et al. [20,21], the influence of the impurity on grain boundary cohesion can be analyzed further by splitting the strengthening energy $\Delta E_{\mathrm{SE}}$ into a chemical and a mechanical contribution, $\Delta E_{\mathrm{SE}}^{c}$ and $\Delta E_{\mathrm{SE}}^{m}$. Formally, this is done by splitting the individual bonding energies (to the grain boundary and to the free surface) in the same way. $E_{B}^{c}$ is the difference between the energy of the relaxed interface with the segregated impurity and the energy of the interface structure from which the impurity has been removed without subsequently relaxing the host lattice, plus the chemical potential of the impurity,

(10) $$\begin{array}{c} E_{B,S}^{c}=E_{\mathrm{tot},S}^{X}-E_{\mathrm{tot},S}^{□}-\mu_{X}. \end{array}$$

*S* labels again the interface (either GB or FS), and the □ indicates the vacant interstitial site which is created when the impurity atom is removed while keeping the host atoms in their positions. The mechanical contribution is the energy which is released when relaxing these atoms and can simply be calculated from

(11) $$\begin{array}{c} E_{B,S}^{m}=E_{B,S}-E_{B,S}^{c}. \end{array}$$

### Grain Boundary Energy

There are different ways to calculate the energy of a grain boundary with segregated elements, with some subtle consequences. Both are presented in the following. The first approach, also used in [15,16], defines the grain boundary energy as the difference between the energy of a supercell, which contains the grain boundary with or without segregated elements, and an equivalent cell with the perfect Fe bulk. To preserve the number of atoms, the chemical potentials of the elements are subtracted as well.

(12) $$\begin{array}{ccc} \gamma_{\mathrm{GB}}^{nC,mH} & = & \frac{E_{\mathrm{tot},\mathrm{GB}}^{nC,mH}-E_{\mathrm{tot},\mathrm{bulk}}-n\mu_{C}-m\mu_{H}}{2A} \end{array}$$

This means that the change in grain boundary energy due to the segregated elements equals the *solution energy* of the elements at the interface (compare Equation (3)):

(13) $$\begin{array}{ccc} E_{\mathrm{sol},\mathrm{GB}}^{aC,bH} & = & \frac{2A}{N}\left[\gamma_{\mathrm{GB}}^{nC,mH}-\gamma_{\mathrm{GB}}^{(n-a)C,(m-b)H}\right]. \end{array}$$

In the alternative formulation of the grain boundary energy, using a bulk supercell with the same number of interstitial atoms,

(14) $$\begin{array}{ccc} \gamma_{{\mathrm{GB}}^{-}}^{nC,mH} & = & \frac{E_{\mathrm{tot},\mathrm{GB}}^{nC,mH}-E_{\mathrm{tot},\mathrm{bulk}}^{nC,mH}}{2A}, \end{array}$$

the change in grain boundary energy equals the *segregation energy* (Equation (4)). Physics based arguments can be found for both [22], but the latter formulation has the drawback that in the typical supercell size in DFT calculations the local concentration in the bulk is artificially high, and the GB energy is thus underestimated, as can be seen in Table 1.

## 3. Computational Details

**Figure 1:**
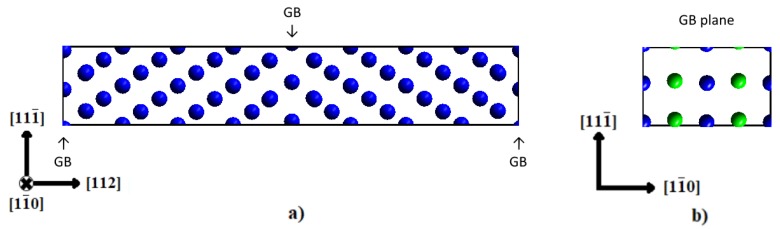
(**a**) Simulation supercell with 24 crystallographic layers parallel to the (112) GB plane which results in 96 Fe atoms. The segregation sites for interstitial atoms at the GB are not visible in this projection, since they are underneath the Fe atoms along the $\left[1\bar{1}0\right]$ direction. (**b**) Grain boundary cross-section with the four interstitial segregation sites marked as green atoms.

The grain boundary supercell is shown in Figure 1. It was created using the orthogonal unit cell vectors $\left[11\bar{1}\right]\cdot a_{0}$, $4\cdot \left[112\right]\cdot a_{0}$, and $2\cdot \left[1\bar{1}0\right]\cdot a_{0}$. The bcc Fe lattice constant obtained from an optimization of the unit cell is a_0_ = 2.83 Å. Thus, the grain boundary area in the cell is 2 × 39.31 Å^2^. In this set-up, up to four interstitial atoms can be placed in each GB plane. The interstitial sites correspond to octahedral sites, with an expanded axis along the $\left[1\bar{1}0\right]$ direction [16]. In the reference bulk structure, the atoms were also placed in octahedral sites. With the given supercell size, the areal density of segregating elements can be varied from 0 to 0.1 atoms/Å^2^.

DFT calculations were carried out using the Vienna Ab-initio Simulation Package (VASP) [23,24]. The exchange and correlation effects were treated within the generalized gradient approximation in the formulation of Perdew, Burke, and Ernzerhof [25], and the projector augmented-wave (PAW) [26,27] method was applied to describe valence–core interactions using an energy cut-off of 450 eV for the basis set. For the sampling of the Brillouin zone, a 4×2×4 Monkhorst–Pack type [28] mesh was used. These convergence parameters were found to be sufficient to obtain converged interface energies in a previous study [16]. Magnetism was considered in a scalar relativistic fashion, i.e., the net magnetic moments are a result of different occupation of majority and minority spin bands in a spin-polarized DFT calculation. The excess volume, i.e., the expansion perpendicular to the GB plane was optimized for each configuration of segregating atoms. The results for the most favorable arrangements of atoms for each C/H content are listed in Table 1.

The reference chemical potentials for calculations of grain boundary and solution energies were $\mu_{C}$ = −9.091 eV for C in the diamond structure, and $\mu_{H}=-3.384$ eV for H in a H_2_ molecule in vacuum.

## 4. Results

### 4.1. Initial Grain Boundary

The optimization of the pure Fe GB leads to an excess length of 0.35 Å and a grain boundary energy of 0.44 J/m^2^, in good agreement with the results of other DFT studies of Σ3(112) STGB, namely 0.43 J/m^2^ [29], 0.46 J/m^2^ [16], and 0.47 J/m^2^ [30]. Note that for the pure Fe STGB two translation states exist, with an small energy difference of 0.02 J/m^2^ [16]: a mirror-symmetric state, as shown in Figure 1, and one with a small shift along the $\left[11\bar{1}\right]$ direction, which was also observed by Bristowe et al. [31]. For the pure GB, the latter is lower in energy, but, once C is present at the interface, this structure becomes unstable and relaxes back to the mirror-symmetric one [16]. The mirror symmetry is maintained when C is replaced by H and the atomic positions are relaxed. Thus, the symmetric structure was kept throughout this study. In the next step, the most favorable segregation patterns at the GB after excess volume optimization were determined. When carbon and hydrogen both cover half of the four possible segregation sites, three possibilities appear: the same species of impurity aligning along the tilt axis, $\left[1\bar{1}0\right]$, along its perpendicular direction, $\left[11\bar{1}\right]$, or along the diagonal, $\left[20\bar{1}\right]$. In Table 2, one can see that after optimization the pattern with the lowest energy and least excess volume is the one in which impurities of the same species align along the tilt axis $\left[1\bar{1}0\right]$. This pattern was thus chosen as the starting point for the calculation of the WoS for this composition.

### 4.2. Solution Energy

**Figure 2:**
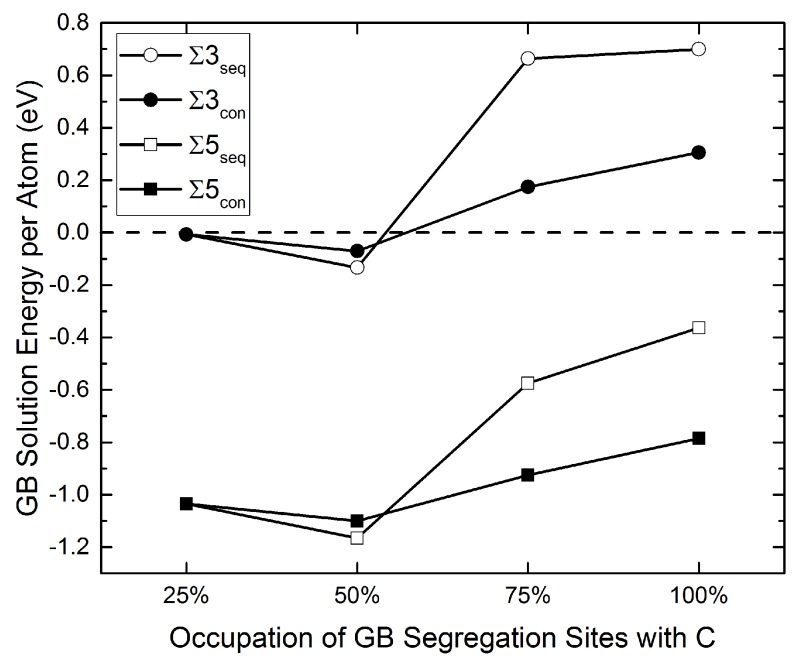
Solution energy of C at different STGBs (compare Equation (3)). The values for the Σ5 STGB (squares) are taken from [15], those for the Σ3 (circles) are from this work. Open symbols denote sequential segregation and full symbols indicate concurrent segregation.

In Figure 2, the solution energy of carbon at the Σ3(112) GB (this work) can be compared to the one at the Σ5(310) GB (from [15]). It is striking that, despite the different GB structures, the solution energy of carbon actually follows the same trend in both grain boundaries, as already claimed in [16]. The whole energy profile of segregation at the Σ3 GB is shifted by approximately 1 eV comparing to that of the Σ5 GB.

### 4.3. Co-segregation of C and H

**Figure 3:**
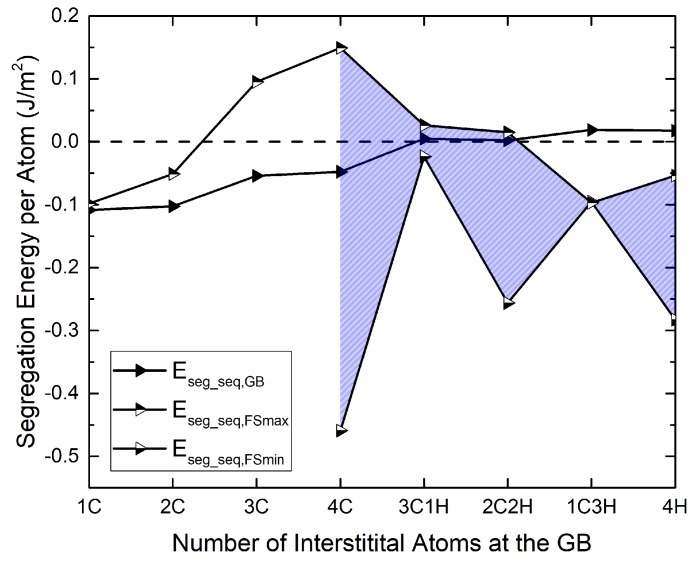
Segregation energies of interstitials at the Σ3(112) GB and the (112) surface with step-wise addition of C and subsequent replacement with H in a sequential scenario (Equations (6) and (7)). The surface segregation energies are the average value for the two surfaces that arise upon separation of the GB. The two different versions (FSmin and FSmax) of the surface segregation energies result from different partitioning patterns (see Table 3).

**Figure 4:**
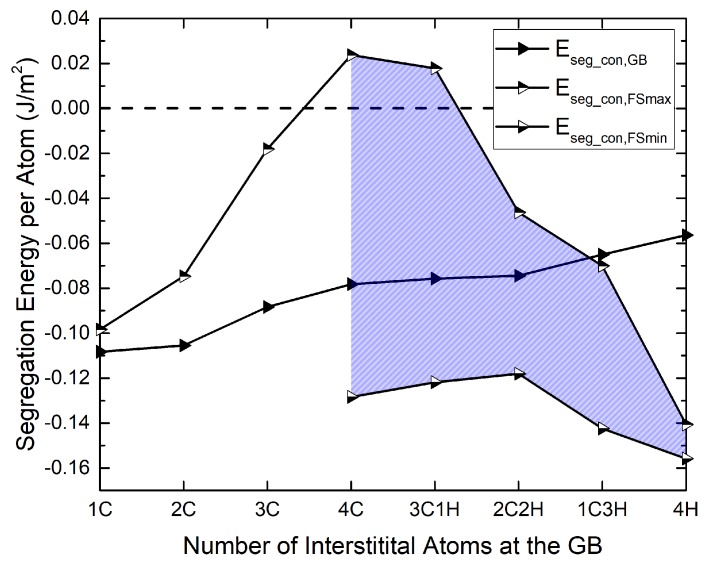
Segregation energies of interstitials at the Σ3(112) GB and the (112) surfaces for different C and mixed C/H contents in a concurrent scenario (Equation (5)). The surface segregation energies are the average value for the two surfaces that arise upon separation of the GB. The two different versions (FSmin and FSmax) of the surface segregation energies result from different partitioning patterns (see Table 3).

After saturating the GB with C, C is replaced by H step by step, and the segregation energy is evaluated in the concurrent as well as in the sequential scheme. The results are shown in Figure 3 and Figure 4 (full symbols). The sequential segregation energies in Figure 3 show that, while they are all negative, they keep increasing as the C concentration increases and even become positive as C is replaced by H. According to these results, a full occupation with H is rather unfavorable.

The comparison with the concurrent scenario, Figure 4, shows the influence of the reference structure: if all elements are assumed to segregate at the same time, the segregation energy, which is now referred to the pure Fe GB, remains negative throughout. However, the full occupation with H remains the most unfavorable condition. Note that, in contrast to the findings of Tahir et al. [15] for the Σ5 grain boundary, no “co-segregation” effect is found, in the sense that a combination of C and H is never lower in energy than C alone at the Σ3 GB.

Before obtaining the work of separation, the segregation of C and H to the (112) surface was investigated. The minimum and maximum segregation energies are also shown in Figure 3 and Figure 4. They are the result of a scan of all possible surface partition patterns, which is presented in the next section.

### 4.4. Partition Patterns and Work of Separation

For a full occupation of available segregation sites, the areal density of segregating elements at the Σ3 coherent twin boundary is roughly 0.1 atoms/Å^2^, i.e., above the critical density 0.04 atoms/Å^2^, which was identified by Wang et al. [16], from which an interaction among the interstitial atoms should be expected. This interaction can lead to pronounced differences in the energy of different partitioning of the elements to and arrangements on the surfaces. Therefore, the partition patterns at the free surface are analyzed in detail in the following.

**Figure 5:**
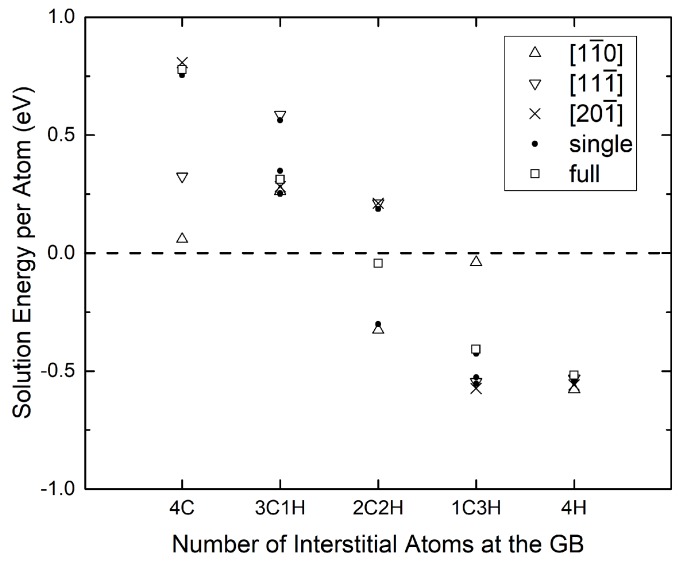
Average solution energy per atom of different C/H combinations at the (112) surfaces cleaved from the Σ3 STGB. The x axis label refers to the C/H content at the GB; after cleavage, the atoms are distributed on the two surfaces in different fractions and arrangements (see Table 3).

At the free surface, the number of possible partitions and arrangements is 32 in total, as shown in Table 3. Their solution energies at the free (112) surface, $E_{\mathrm{sol},\mathrm{FS}}^{nC,mH}$, are shown in Figure 5. All possibilities were considered. They lead to the variation of surface segregation energies in Figure 3 and Figure 4, which represent the results for the most and least favorable sites. Note that the x-axis label marks the corresponding grain boundary configuration. The concentration of C or H on one of the surfaces can only be a fraction of the GB content. A close analysis of Figure 5 shows that the most beneficial configurations, which lead to the maximal reduction of work of separation, are those where the interstitial atoms of the same species align along the direction of tilt axis $\left[1\bar{1}0\right]$.

A further step of optimization of the positions of C and H was taken by assuming that the interstitial atoms could diffuse into the surface layer. The initial, rigid cleavage of the grain boundary layer produces two free surfaces, one of which contains the grain boundary Fe atoms (plus some segregants), while the other one holds only the segregants, which are now situated slightly above the second Fe surface (and remain there even upon relaxation of the atomic positions). To mimic diffusion, which could be triggered by the release of surplus elastic energy, or by thermal activation, these atoms were “nudged” into the surface layer, by placing them into the nearest surface sites and relaxing the atomic positions again. In the case of all patterns in which the species align along the tilt axis, nudging H turns out to be an exothermic process. However, it is known that for the diffusion into the stable tetrahedral site via an octahedral site a rather high energy barrier has to be overcome [32]. For full C occupation, the equivalent nudged state for a C atom is 0.94 eV per atom higher in energy than the “as cleaved” position, rendering the diffusion process rather unlikely.

**Figure 6:**
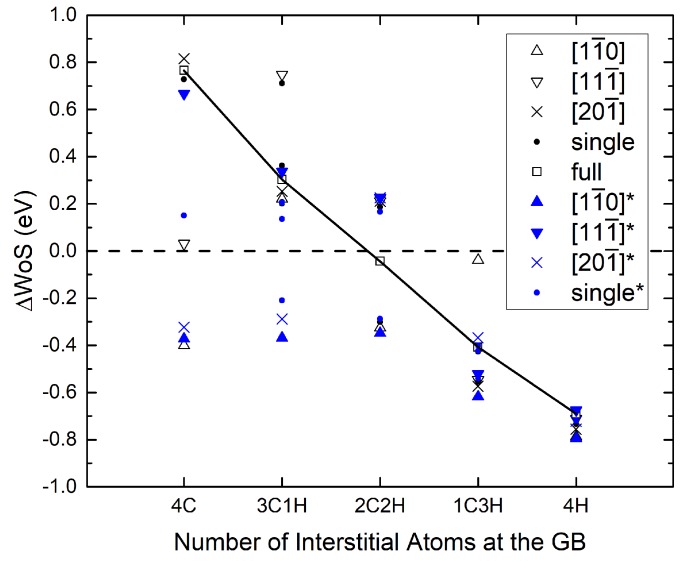
Difference of work of separation of the Σ3 GB for different compositions. ΔWoS corresponds to the strengthening energy (Equation (9)). The different values for each composition arise from different partitioning and arrangement of the atoms on the two surfaces (see Table 3). Values obtained after nudging the interstitial atom from the position on top of the surface into the first surface layer are colored in blue.

Finally, the detailed dependence of the difference in work of separation on the surface configurations can be seen in Figure 6; the nudged sites are also included in blue color and can be compared with the “as cleaved” cases in black color. The black line connects the partition patterns for which all elements are on one free surface, leaving the other surface to be pure Fe.

**Figure 7:**
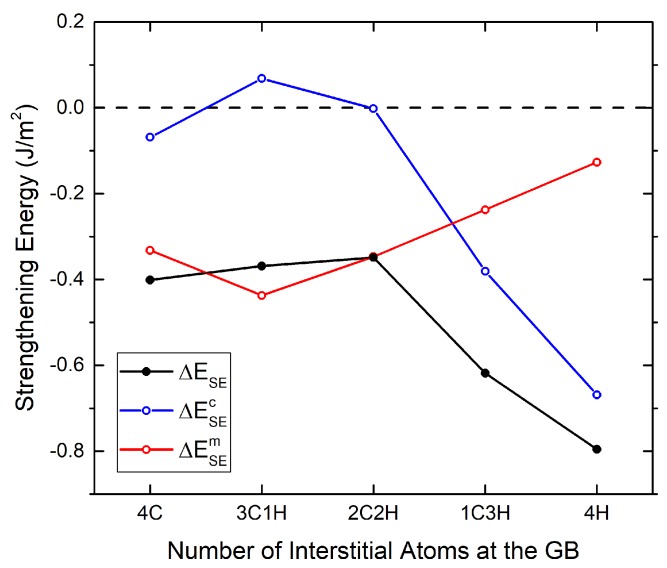
Chemical and mechanical contributions to the strengthening energy of different combinations of C and H atoms at the GB. Positive (negative) values indicate an increase (a decrease) in cohesion.

Since an alignment of chemical species along the tilt axis always exhibits the lowest ΔWoS, the chemical and mechanical calculation for this partition pattern for all compositions is analyzed further (see Figure 7). In contrast to the findings for the Σ5 GB [15], C also shows a negative mechanical contribution to the binding energy. This could be due to the more close-packed structure of the Σ3 GB. The absolute value of the mechanical contribution decreases, as C is replaced by the smaller H atoms, and the reduction in WoS as well as binding energy is dominated by the negative chemical contribution.

## 5. Discussion

In the study of Tahir et al. [15] of a Σ5 STGB, it was shown that a co-segregation of H and C is a possible explanation for H embrittlement. It was based on the insight that a grain boundary in an Fe-C alloy would always be saturated with C, and hence this state should be the reference state to investigate co-segregation with H. Here, this viewpoint is validated for the $\Sigma 3\left(112\right)\left[1\bar{1}0\right]$ STGB, leading to the following observations: independently of the segregation scenario—sequential or concurrent—the configurations with C only have the lowest segregation energies. The substitution of C with H increases the segregation energy, but, in the case of the concurrent picture, the segregation energy remains negative, which means a combined segregation of C and H to the GB is possible, at least from a thermodynamic point of view.

This interpretation is supported by the grain boundary energies (Table 1). The lowest grain boundary energy in this study was obtained for a full occupation with H, if the solution energy of the element is included in the interface energy (Equation (12)), but it is the grain boundary fully occupied by C, if the reference system is a bulk cell of the same composition (with artificially high concentration of interstitials) (Equation (14)). The true optimal configuration should lie somewhere in between these two configurations.

For the process of splitting the grain boundary into two free surfaces, different partition patterns of C and H were taken into account. A large spread in energies is observed for four and three C atoms a the GB, which can be attributed to the long-range C–C interaction. The “corridor” between the most and least energetically favored partition patterns leads to a whole range of possible values for the work of separation, and hence different predictions for the influence of the elements on GB cohesion. Even a full coverage of carbon would weaken the grain boundary, if the C atoms on the surfaces align along the former direction of the tilt axis, [1$\bar{1}$0].

Furthermore, not only the “as cleaved” surfaces with relaxation of ions were considered, but also “nudged” configurations, which emerge if the segregating atom can move from a bridge-like position into the surface layer during the decohesion process. For both elements, H and C, configurations can be found that are lower in energy than those in which all atoms are on the surface. However, the energy barrier for these diffusion processes at the surfaces were not determined in this work. Using literature values for bulk diffusion of around 0.13 eV for H and 1 eV for C [32,33] as a an estimate, the C subsurface position becomes less likely, while the one for H should be expected to occur. This implies that most detrimental cases for 3C1H composition in Figure 6 are not likely to happen, while they are for 1C3H.

The dominating mechanical contribution to the binding energy difference at full carbon coverage, together with its decrease as the fraction of H is increased, suggests that there is an elastic interaction between the C atoms, which is not present between the C and H, or the H atoms. Accordingly, the narrowing of the “corridor” in the work of separation is accompanied by an increasing chemical contribution to the binding energy difference.

Finally, one should mention that for a full characterization of grain boundary cohesion, the WoS is not sufficient, but also the fracture stress under tensile load should be considered [15]. Concepts to take into account the change in H concentration during separation exist for single crystals [34,35], and could probably be extended to grain boundaries. The study at hand, however, shows that the variation of surface configurations, i.e., of the final state of separation, should also be taken into account.

## 6. Summary and Conclusions

In this study, the co-segregation of C and H to the $\Sigma 3\left(112\right)\left[1\bar{1}0\right]$ STGB in bcc Fe was investigated. Compared to the same process at the Σ5(310) STGB [15], the solution energies at the interface are rather high, probably due to the more close-packed structure of the GB. Qualitatively, however, they follow the same trend with C content. The first C atom has the most negative segregation energy at the grain boundary, while the following addition of carbon atoms and also the subsequent substitution of C with hydrogen atoms raise the segregation energy. For the separation process, which splits the GB into two free surfaces, two scenarios can be imagined: in thermodynamic equilibrium, when C and H have enough time to diffuse between the two surfaces during the separation process, the interstitial atoms would always assume the most favorable distribution on the two surfaces. In this scenario, a full coverage of the Σ3 grain boundary with C would actually reduce the WoS below the value of the pure Fe grain boundary, which is expressed in terms of a negative strengthening energy. In a non-equilibrium scenario, if higher-energy partitioning to the free surfaces is allowed, both positive and negative strengthening energies occur, also for all co-segregated cases. A full occupation of segregation sites with H, however, always leads to a reduction of the WoS, independent of the final distribution of H on the free surfaces.

The analysis of the mechanical and chemical contribution of the strengthening energy indicates that the elastic contribution dominates at high C contents and causes a large energy variation with varying partitioning patterns of C on the free surfaces. At higher H content, the chemical contribution dominates, and this variation is smaller. The overall effect on interface cohesion is always negative in these cases.

The study at hand shows that it is difficult to make a general statement on the influence of interstitial elements on grain boundary cohesion in metals, based on the strengthening energy or change in work of separation, especially if the elements exhibit long-range interactions. Instead of taking into account only a single surface configuration for ab initio calculations of the work of separation, the evaluation of an ensemble average (which can quickly become computationally demanding) is recommended.

## Figures and Tables

**Table 1:** Excess length of the GB (i.e., the excess volume divided by the GB area) and GB energy for different compositions. The impurity concentration at the grain boundary is given in percentage of all available segregation sites (four in total). $\gamma_{\mathrm{GB}}$ refers to the GB energy calculated according to Equation (12) (including the solution energy), $\gamma_{{\mathrm{GB}}^{-}}$ is the GB energy according to Equation (14).

Composition	Excess Length (Å)	$\gamma_{\mathrm{GB}}$ (J/m^2^)	$\gamma_{{\mathrm{GB}}^{-}}$ (J/m^2^)
pure Fe bulk	0.00	0.00	0.00
pure Fe GB	0.35	0.44	0.44
25%C GB	0.63	0.44	0.22
50%C GB	0.87	0.38	0.02
75%C GB	1.06	0.65	−0.09
100%C GB	1.21	0.94	−0.19
75%C + 25%H GB	1.10	0.64	−0.17
50%C + 50%H GB	0.94	0.38	−0.16
25%C + 75%H GB	0.83	0.35	−0.08
100%H GB	0.67	0.29	−0.01

**Table 2:** Excess volume and total energy of supercells with 50%C + 50%H coverage at the Σ3 GB. The first column indicates the direction of alignment of two atoms of the same species.

Alignment	Excess Length (Å)	E_tot_ (eV)
$\left[11\bar{1}\right]$	0.97	−858.90
$\left[1\bar{1}0\right]$	0.94	−860.04
$\left[20\bar{1}\right]$	0.97	−859.97

**Table 3:** Partition patterns of grain boundaries and free surfaces with different impurity concentrations. In the left column of the table are the grain boundary arrangement patterns; in the right column are the corresponding various partition patterns on its free surfaces. The signs in the bottom-right corners indicate the direction of alignment of atoms of the same species: Δ: [1$\bar{1}0$]; ▿: $\left[11\bar{1}\right]$; ×: $\left[20\bar{1}\right]$. • indicates that only one vacancy is left and □ indicates that no vacancy is left.

Segregants	Partition on One Free Surface as Δ*WoS* Increases
$\left[\begin{array}{cc} C & C\\ C & C \end{array}\right]$	$[\begin{array}{cc} C & C\\ ◼ & ◼ \end{array}]\begin{array}{c} ▵ \end{array}[\begin{array}{cc} C & ◼\\ C & ◼ \end{array}]\begin{array}{c} ▿ \end{array}[\begin{array}{cc} C & C\\ C & ◼ \end{array}]\begin{array}{c} • \end{array}[\begin{array}{cc} C & C\\ C & C \end{array}]\begin{array}{c} □ \end{array}[\begin{array}{cc} ◼ & C\\ C & ◼ \end{array}]\begin{array}{c} \times \end{array}$
$\left[\begin{array}{cc} C & C\\ C & H \end{array}\right]$	$[\begin{array}{cc} C & C\\ C & ◼ \end{array}]\begin{array}{c} • \end{array}[\begin{array}{cc} C & C\\ ◼ & H \end{array}]\begin{array}{c} • \end{array}[\begin{array}{cc} C & C\\ ◼ & ◼ \end{array}]\begin{array}{c} ▵ \end{array}[\begin{array}{cc} C & ◼\\ ◼ & H \end{array}]\begin{array}{c} \times \end{array}[\begin{array}{cc} C & C\\ C & H \end{array}]\begin{array}{c} □ \end{array}[\begin{array}{cc} ◼ & C\\ C & H \end{array}]\begin{array}{c} • \end{array}[\begin{array}{cc} C & ◼\\ C & H \end{array}]\begin{array}{c} • \end{array}[\begin{array}{cc} C & ◼\\ C & ◼ \end{array}]\begin{array}{c} ▿ \end{array}$
$\left[\begin{array}{cc} C & C\\ H & H \end{array}\right]$	$[\begin{array}{cc} C & C\\ ◼ & ◼ \end{array}]\begin{array}{c} ▵ \end{array}[\begin{array}{cc} C & C\\ H & ◼ \end{array}]\begin{array}{c} • \end{array}[\begin{array}{cc} C & C\\ H & H \end{array}]\begin{array}{c} □ \end{array}[\begin{array}{cc} ◼ & C\\ H & H \end{array}]\begin{array}{c} • \end{array}[\begin{array}{cc} ◼ & C\\ H & ◼ \end{array}]\begin{array}{c} \times \end{array}[\begin{array}{cc} C & ◼\\ H & ◼ \end{array}]\begin{array}{c} ▿ \end{array}$
$\left[\begin{array}{cc} H & C\\ H & H \end{array}\right]$	$[\begin{array}{cc} ◼ & C\\ H & ◼ \end{array}]\begin{array}{c} \times \end{array}[\begin{array}{cc} H & C\\ ◼ & H \end{array}]\begin{array}{c} • \end{array}[\begin{array}{cc} H & C\\ H & ◼ \end{array}]\begin{array}{c} • \end{array}[\begin{array}{cc} H & ◼\\ H & ◼ \end{array}]\begin{array}{c} ▿ \end{array}[\begin{array}{cc} H & ◼\\ H & H \end{array}]\begin{array}{c} • \end{array}[\begin{array}{cc} H & C\\ H & H \end{array}]\begin{array}{c} □ \end{array}[\begin{array}{cc} ◼ & C\\ H & H \end{array}]\begin{array}{c} • \end{array}[\begin{array}{cc} H & C\\ ◼ & ◼ \end{array}]\begin{array}{c} ▵ \end{array}$
$\left[\begin{array}{cc} H & H\\ H & H \end{array}\right]$	$[\begin{array}{cc} H & H\\ ◼ & ◼ \end{array}]\begin{array}{c} ▵ \end{array}[\begin{array}{cc} ◼ & H\\ H & ◼ \end{array}]\begin{array}{c} \times \end{array}[\begin{array}{cc} H & H\\ H & ◼ \end{array}]\begin{array}{c} • \end{array}[\begin{array}{cc} H & ◼\\ H & ◼ \end{array}]\begin{array}{c} ▿ \end{array}[\begin{array}{cc} H & H\\ H & H \end{array}]\begin{array}{c} □ \end{array}$
